# Comparative analyses of whole-genome protein sequences from multiple organisms

**DOI:** 10.1038/s41598-018-25090-8

**Published:** 2018-05-01

**Authors:** Makio Yokono, Soichirou Satoh, Ayumi Tanaka

**Affiliations:** 1 Institute of Low Temperature Science, Hokkaido University, Sapporo, 060-0819 Japan; 2 Nippon Flour Mills Co., Ltd., Innovation Center, Atsugi, 243-0041 Japan; 3 Graduate School of Life and Environmental Sciences, Kyoto Prefectural University, Kyoto, 606-8522 Japan

## Abstract

Phylogenies based on entire genomes are a powerful tool for reconstructing the Tree of Life. Several methods have been proposed, most of which employ an alignment-free strategy. Average sequence similarity methods are different than most other whole-genome methods, because they are based on local alignments. However, previous average similarity methods fail to reconstruct a correct phylogeny when compared against other whole-genome trees. In this study, we developed a novel average sequence similarity method. Our method correctly reconstructs the phylogenetic tree of *in silico* evolved *E*. *coli* proteomes. We applied the method to reconstruct a whole-proteome phylogeny of 1,087 species from all three domains of life, Bacteria, Archaea, and Eucarya. Our tree was automatically reconstructed without any human decisions, such as the selection of organisms. The tree exhibits a concentric circle-like structure, indicating that all the organisms have similar total branch lengths from their common ancestor. Branching patterns of the members of each phylum of Bacteria and Archaea are largely consistent with previous reports. The topologies are largely consistent with those reconstructed by other methods. These results strongly suggest that this approach has sufficient taxonomic resolution and reliability to infer phylogeny, from phylum to strain, of a wide range of organisms.

## Introduction

The reconstruction of phylogenetic trees is a powerful tool for understanding organismal evolutionary processes. Molecular phylogenetic analysis using ribosomal RNA (rRNA) clarified the phylogenetic relationship of the three domains, bacterial, archaeal, and eukaryotic^1^. In addition to rRNA, various orthologous genes have been used for reconstructing phylogenetic trees of life. However, the level of phylogenetic resolution allowed by single genes, particularly at the basal level of the Tree of Life, is insufficient owing to the saturation of nucleotide or amino acid substitutions. One solution to overcome this problem is to use concatenated orthologous genes^2,3^. However, this method is difficult to apply to distantly related organisms and viral genomes, because genes are frequently horizontally transferred^4^, and the number of vertically inherited orthologs shared by distantly related organisms is limited^5^. It is reasonable to use orthologs to elucidate species evolution; however, organismal evolution has occurred not only through the molecular evolution of orthologs, but also through the evolution of non-orthologous genes, because not only orthologous genes but also non-orthologous genes (species specific genes) contribute to the identity of organisms. However, this information is not included in alignment-based phylogenies of orthologous genes.

Another approach is to reconstruct a phylogenetic tree based on whole-genome sequences^6–8^. An enormous number of complete genome sequences are now available. This enables the reconstruction of whole-genome phylogenies for a large number of organisms. Several tree reconstruction methods have been used with complete genomes^9–11^, including gene order, gene content, nucleotide composition, metabolic pathway reaction content, and single-nucleotide polymorphism analyses. These approaches use hidden evolutionary information, not available in single-gene or concatenated gene phylogenetic analyses. However, all these methods have their own limitations, including low resolution and reliability^8,12^. The primary problem with most whole-genome phylogenies is that they are based on alignment-free methodology, which does not incorporate standard molecular evolutionary concepts, although the reconstructed phylogenies are similar to sequence-based trees in many cases^8^. Although whole-genome phylogenetic approaches have many limitations, these methods should be further developed, particularly because the methods can potentially use hidden evolutionary information that cannot be incorporated into alignment-based phylogenetic analyses.

Average sequence similarity methods have been proposed for the reconstruction of whole-genome trees^13–15^. In contrast to other whole-genome tree methods, these average similarity methods use aligned sequence information in which evolutionary distances are calculated from the BLAST bit score^16^ of best-matched pairs. However, this approach has been criticized from various viewpoints:^8^ (1) The approach uses local alignments (BLAST)^16^, instead of global alignment. (2) The approach does not include standard molecular phylogenetic concepts. (3) Many of these methods use reciprocal best matches in which non-orthologous genes are included, thereby introducing noise.

The major problem concerning average sequence similarity approaches is the inclusion of non-orthologous genes, and, in fact, whole-genome trees are improved by excluding non-orthologous genes from the analyses^14^. However, it should be noted that many genes have evolved from ancestral non-orthologous genes through gene duplication followed by divergence processes, and have obtained new functions on these paths. For this reason, non-orthologous gene information should be included to better understand genomic evolution. However, the inclusion of non-orthologous genes in alignment-based phylogenetics is a challenging approach. In a previous report, we developed a whole-proteome method using average sequence similarities that includes non-orthologous gene pairs^17^. The phylogenetic tree of photosynthetic organisms reconstructed by this new method is similar to that of trees previously reported. However, it is not evident whether this method can be applied to a Tree of Life including all three domains. In this report, we have modified our previous method and succeeded in automatically reconstructing a Tree of Life including 1,087 species encompassing all three domains of life, Bacteria, Archaea, and Eucarya. Our phylogeny has high resolution and reliability for both closely and distantly related organisms, despite the exclusion of all human decisions for the selection of organisms and genes. We also show that the inclusion of non-orthologous genes improves the resolution and reliability of phylogenetic trees compared with trees generated by methods that only use reciprocal best hit pairs. Furthermore, our tree supports the three-domain Tree of Life topology.

## Results

### *In silico* evolution of *E*. *coli* proteome and phylogenetic analysis

**Figure 1:**
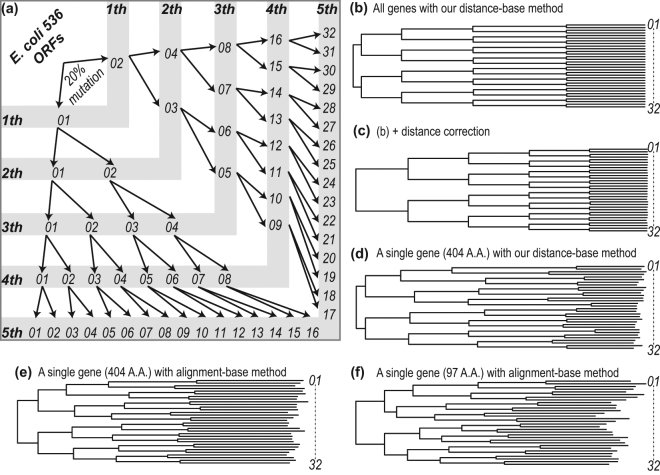
(**a**) Artificial evolution of all open reading frames from *Escherichia coli* 536. Thirty-two genomes after the fifth generation were used to build the phylogenetic trees shown in b–f. (**b**,**c**) Reconstructed trees using all genes evolved *in silico* using our average sequence similarity method. (b) Distance matrix was constructed with *D*. (c) Distance matrix was constructed with *C*. (**d–f**) Reconstructed trees using a single gene. (d) Tree reconstructed from mutated ECP_0844 genes (404 amino acid length) using our average sequence similarity method developed in this study. (e) Similarity dendrogram constructed from mutated ECP_0844 genes, or (f) mutated ECP_0843 genes (97 amino acid length) using the multiple sequence alignment program ClustalX v. 2.1^19^.

We first applied *in silico* evolution to the *E*. *coli* 536 proteome to evaluate the applicability of our method (Fig. 1a). The *E*. *coli* 536 proteome was randomly mutated (20% mutation of amino acid residues per generation) *in silico*, and a phylogenetic tree of the resulting proteomes was reconstructed using our new average sequence similarity method (Fig. 1b). The tree was correctly reconstructed, reflecting the actual evolutionary process used to generate the true phylogeny. The relationship between branch lengths and generations (mutation time) was then examined in more detail. The lengths of each internal branch per generation are almost all the same except at the initial phase of *in silico* evolution (Fig. 1b). The distance between two strains is less than 0.85, if the two strains have a common ancestor in the third or subsequent generations (SupportingFile1.xls, sheet 3). The distance is greater than 0.95, if the common ancestor is in the second generation. As shown in the saturation curve (Supplementary Fig. 1d, black line), *D* is approximately linearly related to evolution time (mutation value) when the value *D* is lower than 0.85 (*C* < 12, corresponding to ~70% amino acids replacement). These results suggest that *D* can be used as an evolutionary distance, and that if the value *D* is lower than 0.85, then the phylogenetic tree can be inferred as being reliable. The length of each internal branch became the same after correcting the distances (Fig. 1c) using the saturation curve constructed by 10% mutation per generation (Supplementary Fig. 1d, black line).

### Reconstruction of a Tree of Life

Next we reconstructed a phylogenetic tree from the proteomes of hundreds of organisms from all domains of life using our new method. We used the following two criteria to properly evaluate the method: 1) It is well known that the topology of a phylogenetic tree can be dramatically affected by gene and taxon selection, and that the presence of certain genes and/or taxa can sometimes seriously disturb tree topology. To exclude the arbitrariness of our genome selection we used all the genomes available that had been completed as of 2011. This included a wide range of organisms from all three domains of life, Bacteria, Archaea, and Eucarya (SupportingFile1.xls, sheet 4). This criterion will help show whether our average sequence similarity method is applicable to a wide range of organisms. 2) All of the procedures were performed automatically, which completely excludes human factors in tree reconstruction.

**Figure 2:**
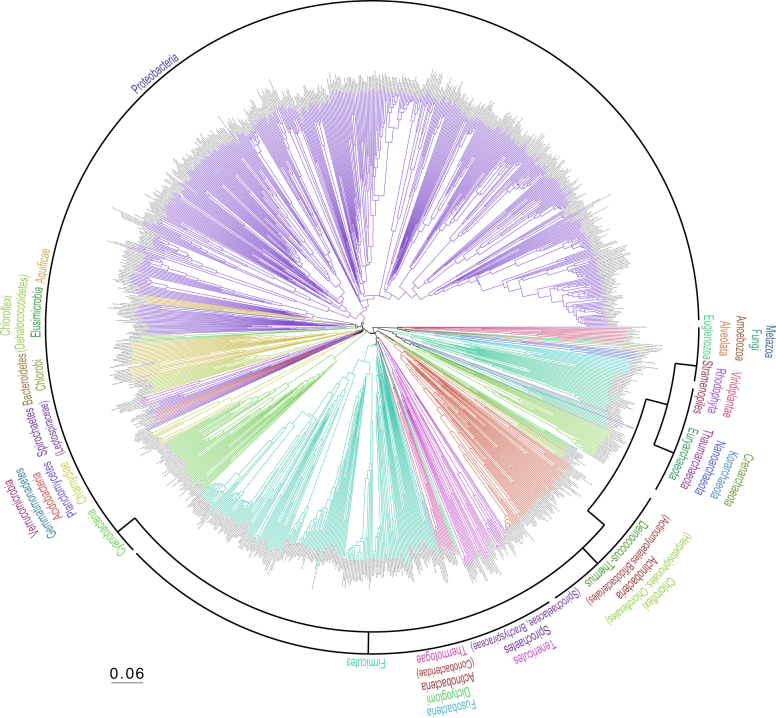
Phylogenetic tree of 1,087 species reconstructed from a comparison of all protein sequences from all the species. Branch colors reflect taxonomic information (division) obtained from the NCBI Website.

**Figure 3:**
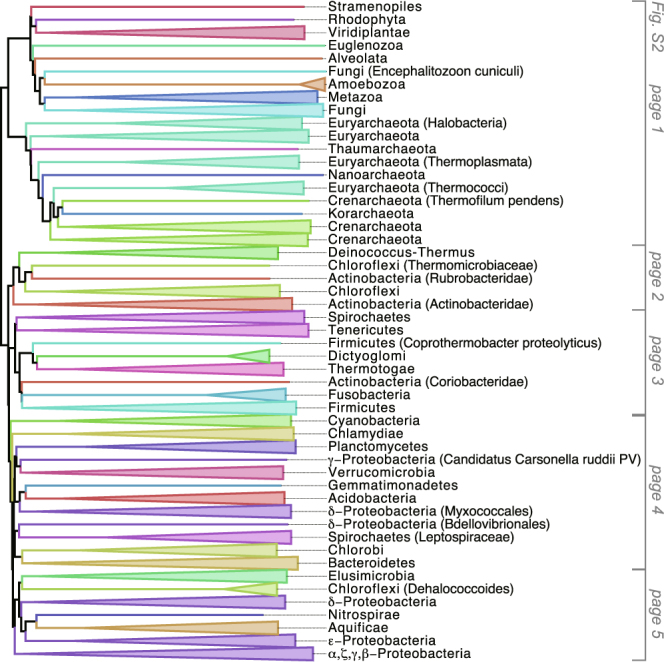
Smaller version of Fig. 2. Species within each clade were collapsed according to taxonomic information (division).

Figures 2 and 3 shows the phylogenetic tree of 31 Eucarya, 62 Archaea, and 994 Bacteria, whereas supplementary Figure 3 shows the same tree, but without two parasites (*Encephalitozoon cuniculi* and *Nanoarchaeum equitans*). The three domains are clearly separated, as reported in other whole genome phylogenies^6,18^. One conspicuous characteristic of our tree is that the total branch lengths from the common ancestor are nearly the same among all of the organisms, resulting in a concentric circle-like structure for the tree. We compared single-gene and the whole-proteome trees from the *E*. *coli* population that we had evolved *in silico* to attempt to explain this phenomenon. The branch lengths of the whole-proteome tree corresponding to the same generation time are exactly identical among various evolutionary lines, which gives rise to a concentric circle like structure as shown in Fig. 1b and c. In contrast, branch lengths of the single-gene tree reconstructed by our new method, or by the alignment-based Neighbor-Joining (NJ) method using a traditional distance matrix corrected for multiple substitutions calculated from the entire alignment^19^, are different between the different evolutionary lines (Fig. 1d–f). This result led to the idea that the branch lengths became similar by averaging the branch lengths of the large number of proteins in the proteome.

This feature was not significantly altered after our saturation correction (Supplementary Fig. 1e), nor by tree reconstruction method (Supplementary Fig. 1f and g).

### Comparison of entire tree topology

We then compared topologies of the three domains generated by our method with previous reports to evaluate our method’s topological robustness. Our tree topology is largely consistent with previous reports that focus on the phylogenetic relationships of restricted groups (see Supplementary Text 1). Therefore, we compared our tree topology with that of other large-scale, multi-gene or entire genome organismal trees. Ciccarelli *et al*. reported a Tree of Life constructed with the maximum likelihood method using concatenated genes^18^; this was used as a benchmark for evaluating our method. For this purpose, we constructed a tree (Supplementary Fig. 4a) using the same organisms as reported by Ciccarelli *et al*. (Fig. 2 and Table S4 in Ciccarelli *et al*.^18^). Ciccarelli’s tree has 163 branching points supported by 80–100% bootstrap values. Among the 163 branching points, 152 (93.3%) are identical to our tree (Supplementary Fig. 4a). This result indicates that our tree is very similar to a Tree of Life constructed by the maximum likelihood method using Ciccarelli’s dataset.

Although comparison with other reports cannot fully evaluate our new approach, because the true Tree of Life is impossible to know, the phylogenetic topology that we recover is quite close to previous reports, for both intra-generic and cross-domain analyses, suggesting that our whole-proteome average sequence similarity approach has high resolution for both closely and distantly related organisms, encompassing Bacteria, Archaea, and Eucarya.

**Figure 4:**
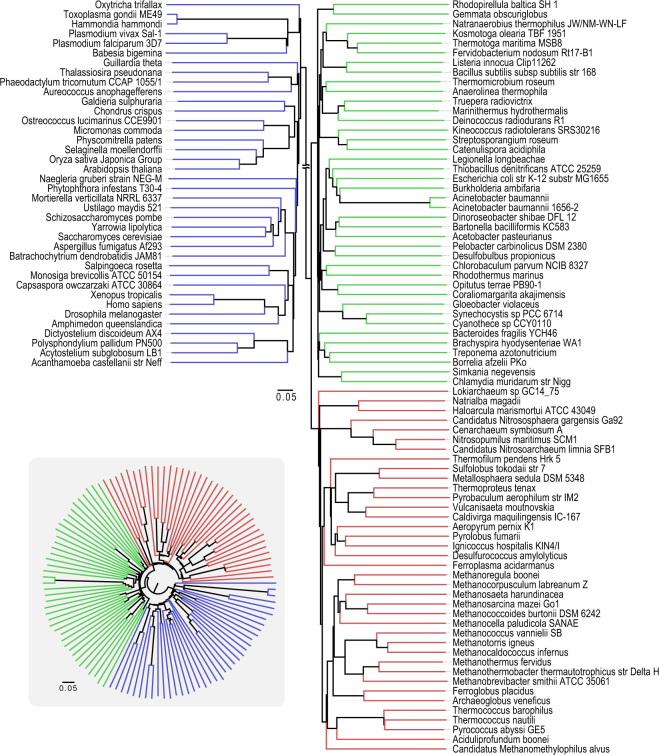
Phylogenetic tree of 116 species reconstructed from a comparison of all protein sequences using the fitch-margoliash method. The 116 species are constructed with random and equal sampling from latest genomes from the three domains^23^. An inset is polar tree layout.

We used all the complete genomes available, as of 2011, to exclude arbitrariness in genome selection (Fig. 2). However, a great number of genome sequences have become available subsequent to 2011. Phylogenetic analyses including uncultivated, newly discovered Archaea phyla suggest the emergence of Eucarya from within Archaea^20–22^, which is different from our tree. One possible reason for this discrepancy is that our tree (Fig. 2) does not include these newly discovered phyla. Another potential factor is our tree contains a much larger number of Bacteria (944) versus Eucarya (31) and Archae (29), and this bias may affect tree topology. Therefore, we constructed a new phylogenetic tree of 106 organisms (Fig. 4) that includes these newly discovered Archaea lineages, and we used a similar number of organisms from each domain, Bacteria (40), Eucarya (38) and Archaea (38)^23^. The three domains remain clearly separated in the new tree, still supporting the three-domain tree of life. Based on these results, it was finally concluded that the three-domain tree of life is supported by our present method. Next, we compared the branching pattern of this tree with recently published trees. In our tree, Lokiarchaeota deeply branched in Archaea, which is consistent with reports which support three-domain tree of life^23,24^ and also consistent with a tree constructed by 45 concatenated genes^25^. In contrast, Lokiarchaeota branches off more recently within the Archaea in the two-domain tree of life constructed by concatenated ribosomal proteins^26^. The branching pattern of Archaea members in our tree is largely consistent with a Bayesian phylogeny using markers including more than ten thousand amino acid positions^27^, except that Halobacteria deeply branch off within the Archaea in our tree (Fig. 4). The branching pattern of Eucarya is similar to an RNA polymerase tree^23^.

### Impact of laterally transferred genes on tree topology

Our method uses all of the genes in the genome, and does not exclude laterally transferred genes in the analyses, in spite of the extensive occurrence of lateral gene transfer throughout the evolution of life and its potentially confounding influence. We used two different approaches to examine the impact of laterally transferred genes on our tree topology.

First, using our *in silico* evolution of the *E*. *coli* 536 genome, we artificially introduced lateral gene transfers between two of the descendants (Supplementary Fig. 4e). The laterally transferred genes were randomly selected. In both cases, the branching pattern did not change when the numbers of the transferred genes was lower than 40% of the total genes. When 50% of the genes were transferred, the branching pattern was changed.

Second, lateral gene transfer was introduced *in silico* between real genomes (Supplementary Fig. 4c and 4d). Lateral genes were randomly selected from *Salmonella* or Cyanobacteria, and added to *E*. *coli* 536 genome. When 10 or 30% of the genes were artificially transferred from *Salmonella* to *E*. *coli* 536, the topology was not changed. However, when 10 or 30% genes were artificially transferred from a distantly related organism (Cyanobacteria) to *E*. *coli* 536, the branching position of *E*. *coli* O157H7 EDL933 was slightly changed, but that of other organisms did not change. The branching position of *E*. *coli* 536 itself, which acquired a large number of laterally transferred genes in the experiment, was not changed at all. Although we could not exactly quantitate the impact of laterally transferred genes on our tree topology, the above two experiments indicate that the tree topology constructed by our method is not largely affected by laterally transferred genes.

## Discussion

Our tree was automatically reconstructed; that is human decisions, such as alignment and selection of genomes (organisms), were completely excluded. *Gloeobacter* is the most deeply rooted cyanobacterium (2.7 Gy ago)^28^, and *E*. *coli* and *Salmonella* diverged about 0.12 Gy ago, with all *E*. *coli* species being more recently diverged^29^. These organisms’ phylogenetic topology was correctly reproduced by our method. Other topologies, including the relationships between eukaryotes and Archaea, are also consistent with previous reports. These results strongly suggest that our new approach has sufficient taxonomic resolution for taxa ranging from domain to strain for a wide range of organisms spanning the Tree of Life. However, drastic decreases in genome size generate incorrect topologies. For example, *Encephalitozoon cuniculi* GB-M1, a parasitic fungus with a greatly reduced genome, about 2,000 protein-coding genes, does not cluster with other Fungi in our analysis. Similarly, *Nanoarchaeum*, the smallest known genome in the Archaea, with only 536 protein-coding genes^30^, is not correctly located in our tree. However, these organisms may have a sufficient number of ORFs to calculate evolutionary distances using our method, because other organisms containing reduced genome sizes (e.g., *Prochlorococcus* with 1,800 protein-coding genes) are well resolved in our analysis, and correct trees have been reconstructed from artificially reduced genomes using the previous version of our method^17^. The incorrect placement of these organisms in our tree suggests that drastic changes in genome sequence have occurred along with a reduction in genome size in these organisms, which is consistent with previous reports^31,32^.

Our phylogenetic analysis of *in silico* evolved *E*. *coli* proteomes shows a linear relationship between generation time and proteome tree branch length when the evolutionary distance *D* is below 0.85. We calibrated the relationship between geologic/evolutionary time and branch length in our Tree of Life using the fossil record and molecular markers (Supplementary Fig. 1 h, red). Branch lengths per year lengthen after 0.6 Gy ago. Branch lengths relate to the degree of genome diversification in our tree, because of the phylogenetic distance calculations we use. Therefore, if the values from the fossil record and molecular markers that we used are correct, some important biological or environmental event such as a rise in atmospheric O_2_ concentration may have occurred around this time (about 0.6 GY ago) that could have facilitated the rapid diversification of the genome. Branch length saturation (Fig. 1b and c) cannot be a major reason for this phenomenon, because similar profiles are also obtained after correction by the saturation factor (Supplementary Fig. 1h, blue).

Another important feature of our tree is that it exhibits a concentric circle like structure. This was not caused by the tree reconstruction method itself, BioNJ^33^, because the same tree structure was made by the NJ method (for all 1,087 species) and the Fitch-Margoliash method (a 721 species subset) as well (Supplemental Fig. 1f and g). This indicates that all the organisms have similar total branch lengths from their common ancestor. This feature might be a typical characteristic of an average similarity tree. However, this topology is not consistent with a report that substitution rates vary significantly between species, and that this variation is associated with species characteristics such as generation times in animals and plants^34^. The evolutionary rates of Bacteria have also been reported to be influenced by generation times^35^. In contrast, branch lengths generated by our present method are almost the same among different lineages, with the exception of some parasites, suggesting that rates of genome evolution are not largely affected by species. We presently cannot answer this discrepancy, and encourage further study.

There are two approaches for the reconstruction of a phylogenetic tree; one is alignment-based, and the other is alignment-free. Whole-genome phylogenetic inference methods, such as gene order and gene content, are alignment-free phylogenies, where sequence information is only used for the annotation of genes^6,8,36^. Traditional alignment-based approaches are impractical for inferring whole-genome phylogenies, because the creation of global, multiple sequence alignments of all the amino acids from or, even more difficult, all the nucleotides of whole-genome sequences for large numbers of even somewhat divergent organisms is presently unrealistic, due to computational limits and complexity. Another alignment-based approach is to create multiple sequence alignments of single or concatenated genes or gene products. However, this method requires human decisions and biases concerning gene and/or organism selection. And, even though it becomes more powerful as more genes are added, it also eventually becomes computationally intractable as the size of the dataset increases.

**Figure 5:**
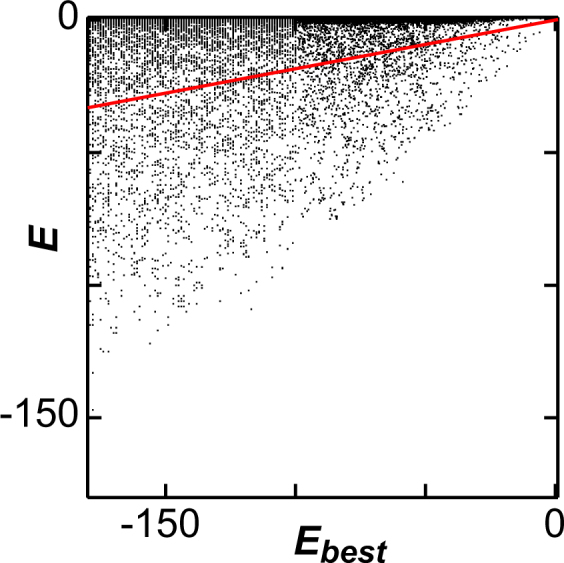
Representation of best-matched proteins on a two-dimensional display. The vertical axes represent the logarithmic *E*-values (**E**) of the best-matched proteins of *Arabidopsis* to the proteins of *Chlamydomonas*. The horizontal axes represent the logarithmic *E*-values of the best-matched proteins of *Arabidopsis* to *Arabidopsis* (***E***_***best***_). In this case, the best-matched proteins are identical to the query proteins. The plot was fitted with a straight line from the origin (red line). We estimated the average of ***T*** from the slope of the line.

Our average sequence similarity method is quite different from these other two alignment-based approaches, because our average sequence similarity method reconstructs a whole-proteome phylogeny based on the local alignments of amino acid sequences. Previously, species trees have been reconstructed by using only orthologs from these local alignments. Some average sequence similarity methods use the mean normalized blastp scores of reciprocal pairs to restrict the phylogenetic analysis to orthologous pairs^14^. A major criticism of this approach is that all orthologous genes may not be correctly defined using only reciprocal best matches^8^. However, this criticism does not apply to our analyses, because we found that the inclusion of non-orthologous gene pairs reconstructs a better topology with our method. In our study we compared phylogenetic trees reconstructed using reciprocal pairs (Supplementary Fig. 1i) with those reconstructed using directional pairs (Fig. 2). A large portion of the reciprocal pairs is expected to be orthologous pairs. In contrast, a large portion of our directional pairs has high *E*-values, around 10^1^ (Fig. 5), indicating undetectable homology between the paired genes. *E*-values of these pairs with undetectable homology were included in the calculation of genomic evolutionary distances in our present study. However, the overall tree topology was more correctly reconstructed using our directed pairs approach than with the reciprocal pairs method. For example, *Cyanidioschyzon merolae*, a Rhodophyte, is most deeply rooted in the eukaryote clade in the tree by reciprocal pairs method. This conclusion is completely against the idea that only orthologous pairs must be used for the average sequence similarity method. Why do directed pairs result in the correct topology? BLAST is used in our new method between all of the individual proteome sequences; therefore, the *E*-values obtained are the expectations that particular proteins are present in the targeted genome. All of the probabilities (*E*-values) are converted to information content scores by conversion to a logarithmic value. Scores of all the pairs are then used for calculating pairwise genomic evolutionary distances. For this reason, our calculated phylogenetic distances describe the probability that genome A evolved from Genome B. Concepts of orthologous genes and function are not included in this calculation process. This is one of the reasons why we use all of the best-matched pairs for calculating genomic evolutionary distances. This calculation process is quite similar to that of calculating protein evolutionary distances from amino acid substitution probabilities (Supplementary Fig. 1a). In the case of traditional phylogenetic analyses, probabilities of amino acid substitutions are converted to scores (e.g. by the PAM250 score matrix) through conversion to logarithmic values, and all these scores are summed to obtain global similarity scores between any two sequences. Over extremely long evolutionary periods genes not only evolve from obviously orthologous genes, but also from genes in which any sign of homology has been lost. This may be another reason why our inclusion of gene pairs with very high *E*-values results in an improved phylogenetic tree. This strategy is quite different from the reconstruction of a species tree by a single or concatenated gene dataset in which the selection of vertically inherited orthologs is indispensable, because the selected genes must have the same evolutionary history as the species. Our method takes into account the fact that organismal evolution occurs not only by the molecular evolution of orthologous genes, but also by the acquisition of species-specific genes. Ideally, this information has to be incorporated into the reconstruction of a species tree. The present approach attempts to include this type of information.

The process of phylogenetic tree reconstruction using any distance matrix method can be divided into two parts: first, the calculation of evolutionary distances, and second, the reconstruction of a phylogenetic tree using those calculated evolutionary distances. Laterally transferred genes are generally considered to cause noise in calculating evolutionary distances. This is particularly true when the phylogenetic tree is constructed by an alignment-based method with either single or concatenated genes datasets. However, the number of detectable, strictly vertically inherited orthologous genes amongst all of the genomes of life is relatively small, with most genes in all of life being laterally transferred and then vertically inherited^18^. Laterally transferred genes do cause noise on the one hand, but also can provide important information, if treated correctly. We tried to evaluate the impact of laterally transferred genes by introducing artificial lateral gene transfer (Supplementary Figs. 4c-4e). Although the impact of laterally transferred genes on the tree topology was not quantitatively evaluated in our experiments, tree topologies were not largely affected by the lateral gene transfer events. This may be an advantage of our method. However, it should be mentioned that the phylogenetic position of *Halobacterium* sp. NRC-1 in our tree is different from that of Ciccarelli *et al*.^18^. This difference may be due to the large number of laterally transferred genes in this archaeal organism. *Halobacterium* sp. NRC-1 has acquired 1,089 genes by lateral gene transfer from the bacterial domain, which implies that 41% of its total genes (1,089/2,630) were laterally transferred. This corroborates our *in silico* experiments, and further demonstrates that a very large number of laterally transferred genes can disturb tree topology^37^. Recently, a list of horizontally transferred gene candidates has been compiled for both Bacteria and Archaea^38^. The exclusion of highly transferred genes from analyses based on such lists is a possible strategy for overcoming the horizontal gene transfer problem. In contrast, horizontally transferred genes are not much of a problem in Eucarya because horizontal transfer is quite limited in that domain^39^. Further study is required concerning the impact of laterally transferred genes. A second unsolved question is whether it is reasonable to allow multiple query genes to pair with the same gene. At present, we have no answer as to whether our procedure is entirely adequate for calculating evolutionary distances, though it does appear to approximate distances well enough to reconstruct acceptable phylogenies.

Distance-based tree topology also depends on the algorithm used to reconstruct a tree from a distance matrix. BioNJ^33^ may have produced a more accurate topology than NJ, because of the huge size of our data set. According to our preliminary calculation with a smaller number of proteomes, the Fitch–Margoliash method, even without the global rearrangement option, can reconstruct a more realistic topology than the NJ method with our data (Supplementary Fig. 1f and g). However, presently the Fitch–Margoliash method cannot be applied to a 1,087 proteome phylogeny due to calculation time constraints, regardless of global rearrangement option.

Recently, extensive research has attempted to elucidate the eukaryote origin, with many reports supporting a two-domain Tree of Life^20–22^. However, questions have been raised concerning this hypothesis^23,24^ because of difficulties in determining a root, and problems with using concatenated genes having independent evolutionary histories. This selection of genes for phylogenetic analysis greatly affects tree topology. Our method attempts to overcome these problems by using all of the protein sequences in a genome for tree reconstruction, but not using a concatenation process. It should be noted that alignment-free whole genome phylogenies that utilize protein domain content and abundance also support the three-domain phylogeny^24^. Improvement of whole genome phylogenetic methods will be required to fully resolve the two versus three-domain issue.

Our present method automatically reconstructs a reasonable Tree of Life. However, theoretical analysis of our method has not been sufficiently undertaken. Further improvements of the method are expected by establishing ways of incorporating various properties of the evolutionary events that lead to extant genomes in the Tree of Life and understanding those theoretical backgrounds.

## Methods

Herein, we propose, develop, and demonstrate a novel average sequence similarity whole-proteome method for reconstructing the Tree of Life. In our new method, phylogenetic distances are calculated by averaging similarities between directional best-hit pairs. Although the method has many problems (a long calculation time, and the limited availability of whole genome species), it is potentially a very powerful tool because it includes local alignments, in spite of being a whole-genome phylogenetic method. Previous average sequence similarity approaches aimed at only using orthologous pairs by employing reciprocal best match pairs with a cut off *E*-value above some particular value, although non-orthologous pairs could not be completely excluded.

Our aim is to automatically reconstruct a Tree of Life while excluding human decision making processes, such as the selection of organisms, determination of gene pairs, construction of distance matrices, and tree reconstruction itself. Instead of reciprocal best match pairs, our method uses the *E*-values of directed best-hit pairs. A large number of non-orthologous pairs are included in these pairs, because many genes are not shared between any two organisms. The procedure follows, in detail.

All of the deduced amino acid sequences from the ORFs of one organism (***X***) are used as query sequences, and blastp searches are performed against all of the amino acid sequences from the ORFs of another organism (***Y***)^40^. For each query ORF (***x***), the *E*-value (***E***) for its best-matched ORF (***y***) is calculated (Supplementary Fig. 1a). If organisms ***X*** and ***Y*** are identical, the best-matched pair is that of the identical ORFs and its *E*-value is referred to as ***E***_*best*_. Next, divide the log-transformed ***E*** by log-transformed ***E***_*best*_, which is referred to as ***T***.

1 $$\frac{Lo{g}_{10}E}{Lo{g}_{10}{E}_{best}}=Lo{g}_{{E}_{best}}E=T$$

This can be transformed as follows:

2 $${({E}_{best})}^{T}=E$$

Here, the *E*-value means the probability that a query sequence appears in an unrelated database sequence, if the size of targeted database is constant. Then, ***E***_*best*_ indicates the probability of an event (***A***) that randomly changes a sequence to ***x*** by coincidence, and ***E*** indicates the probability of an event (***B***) that randomly changes a sequence to the shared sequence between ***x*** and ***y*** by coincidence. Therefore, ***T*** shows the frequency of event ***A*** occurring when event ***B*** occurs once, and **1**−***T*** reflects the relative frequency of the change of the common ancestral sequence of ***x*** and ***y*** to ***x*** (Supplementary Fig. 1b). ***T*** is in the range 0 to 1, and when ***x*** is similar to ***y***, ***T*** nears 1.

All pairs of *Log*_10_***E*** and *Log*_10_***E***_***best***_ corresponding to all ORFs of ***X*** are plotted on a two-dimensional graph, as shown in Fig. 5. We divide the dots into fourteen groups corresponding to the values of ***E***_*best*_ and examine the distribution pattern of ***E***, to examine whether the dots can be fitted as a straight line from the origin. The patterns are very close among the fourteen groups, as shown in Supplemental Fig. 1c. This relationship is observed with all combinations of the two genomes (Supporting File1.xls, sheet 1 and 2). Based on these results, the dots are fitted with a straight line. The straight line from the origin (Fig. 5, red line) is then determined by a least-squares fit analysis. We then use the slope of the line as an average of ***T*** (***T***_*x*→*y*_), and that average is used to estimate a distance between ***X*** and ***Y**** (****D***_*x*↔*y*_). Here, ***T***_*x*→*y*_ is not always the same as ***T***_*y*→*x*_, because proteome sizes and the number of intrinsic genes are different between different organisms. The distance *(****D***_*x*↔*y*_) is approximated by subtracting the average value of ***T***_*x*→*y*_ and ***T***_*y*→*x*_ from 1, and is used for the construction of our distant matrix.

3 $${D}_{x\leftrightarrow y}=1-\frac{{T}_{x\to y}+{T}_{y\to x}}{2}$$

The best-matched genes of ***Y*** are categorized based on a reverse blastp search against ***X***. The best-matched genes of ***Y*** are only used for the ***D*** calculation of the reciprocal tree, when the reverse search gives back the original ***X*** query gene.

Tree topologies are more correctly reconstructed using our directed pairs approach versus reciprocal pairs, indicating that the inclusion of non-orthologous pairs results in a more correct calculation of genomic distances. Our tree topology is nearly consistent with previous reports that include Bacteria, Archaea, and Eucarya. One conspicuous characteristic of this tree is that the total branch lengths from the common ancestor to the tips are almost the same among all the organisms resulting in a concentric circle like structure for the tree. The reliability of this newly developed sequence similarity method was supported by the analysis of *Escherichia coli* proteomes that were evolved *in silico*. A problem with our method is it requires a very long calculation time. For example, the method required almost three years calculation time to obtain the *E*-values for all the pairs from 1,087 proteomes with 24 CPU cores. However, these pairwise BLAST calculations are performed in parallel and, therefore, this time restriction is directly related to the number of CPU cores.

### Data Availability

*E*-values for all the pairs from 1,087 proteomes, which were used for the construction of our distant matrix, are available from the corresponding author on reasonable request. Distance matrices and tree files in FigTree format will be available from FigShare (https://figshare.com/10.6084/m9.figshare.5677705).

## Electronic supplementary material

Supplementary information

Supporting File 1.xls
