# Supplementary material for: Comparative analyses of whole-genome protein sequences from multiple organisms
Source: Sci Rep. 2018 May 1;8:6800. doi: 10.1038/s41598-018-25090-8 (PMC5931523; doi:10.1038/s41598-018-25090-8)
Supplement: Supplementary file 1 — Supplementary information [file 41598_2018_25090_MOESM1_ESM.pdf]

Supplementary information

Manuscript Title:

Comparative analyses of whole-genome protein sequences from multiple organisms

Author names:

Makio Yokono, Soichirou Satoh, Ayumi Tanaka

This file contains Supplementary Methods, SupplementaryText1, and Supplementary Figure 1–5. SupportingFile1.xls is uploaded separately.

Supplementary Methods

1. Sequence data and the BLAST program

All of the ORFs and taxonomic information from 1,087 organisms, which were available in 2011, were retrieved from appropriate Websites (http://www.ncbi.nlm.nih.gov, http://jgi.doe.gov, http://oxy.ciliate.org/, and http://merolae.biol.s.u-tokyo.ac.jp). We obtained pairwise *E*-values for all the ORFs from the 1,087 organisms included in our analysis in an all-versus-all fashion, using the stand-alone blastp program from the BLAST suite ver. 2.2.20 obtained from NCBI website [16], controlled by Mathematica (Wolfram Research Inc., ver. 7.0.1), on three MacPro computers (a total of 24 cores, Apple Inc.). Default parameters and blastp settings were used, as previously described [17,40]. *E-value* cutoffs were set at 10. Resulting pairwise *E*-values ranged between 10^{-180} and10^{0}.

2. *In silico* evolution of genome datasets for the evaluation of saturation effects

We first simulated artificial evolution of all the ORFs from each organism in our study, to evaluate saturation effects (Supplementary Fig. 1d). In the first step, 10% of the amino acids of organisms ***X*** were mutated randomly by Mathematica, and the distance ***D*** was calculated between the original ***X*** and the mutated ***X***. The step was repeated until the distance change was saturated. Then, the saturation curve of ***X*** was used to obtain corrected distances from ***X*** to all the others. We constructed a linear interpolation function from the saturation curve, which converted the distance ***D*** to the repeat count of the 10% mutation ***C***. ***C*** may be proportionate to time when we assumed a constant rate of amino acid mutation, and we used that ***C*** as the corrected distance.

Next, we validated the saturation curves. All of the ORFs from the *E. coli* 536 genome were evolved *in silico* and an evolutionary tree was reconstructed from the descendants using our present method. This time, in one step, 20% of the amino acids were mutated randomly, and 32 artificial strains from the fifth generation were constructed (Fig. 1a). Trees were reconstructed from all genes (Figs. 1b and c), or from single genes (Fig. 1d) using our present method. All trees recovered the correct history of artificial mutation. Furthermore, all branches displayed the same length after applying the distance correction (Fig. 1c) using the *E. coli* 536 genome saturation curve (Supplementary Fig. 1d, black line), which validated the distance correction process.

Previous reports about fossil records and molecular markers [41-53] were used to examine the relationship between time and branch lengths (Supplementary Fig. 1h).

3. Tree reconstruction from all of the protein sequences

Our distance-matrix was constructed as described in NEW APPROACHES. Our whole-proteome

tree of 1,087 species was reconstructed from our distance matrix using BioNJ [33], and drawn and colored (according to taxonomic information obtained from NCBI) using FigTree ver. 1.4.2 (http://tree.bio.ed.ac.uk/software/figtree/). Midpoint rooting was used to root the tree.

For an evaluation of our method, a phylogenetic tree of 187 species was also constructed. The 187 species were selected based on Table S4 in Ciccarelli et al. 2006 [18]. A distance-matrix was constructed as described in Methods. A phylogenetic tree of 187 species was reconstructed from our distance matrix using BioNJ, and drawn using FigTree. The phylogenetic tree of 187 species was compared to the previous report (Fig. 2 in Ciccarelli et al. 2006 [18]). The calculation took 15 days and 10 hours with 20 CPU cores. Theoretically, calculation times could be reduced to one day by using 310 cores, because parallel calculation can be performed independently with each query species.

4. Consensus tree and other demonstration trees

Our consensus trees were constructed using consense, Fitch–Margoliash trees by fitch, and NJ trees by neighbor, all from the PHYLIP package ver. 3.69 [54], controlled by Mathematica. Default parameters and settings were used, except that global rearrangement was performed in fitch. Our 1,087 species are classified into 144 orders by NCBI. One species was randomly selected from each order, and a distance matrix of those 144 species was constructed by our average sequence similarity whole-proteome method. A tree was then built using the Fitch–Margoliash method. This process was repeated 100 times, reconstructing 100 trees. Our consensus tree was built from those 100 trees.

5. Lateral gene transfer during *in silico* evolution of *E. coli* genome datasets

All of the ORFs from the *E. coli* 536 genome were evolved *in silico* as previously described (Fig. 1a). However, at the fourth generation (the evolutionary stage one before the end of our simulation), 0–70% of the genes from species "4th01" were randomly selected and laterally transferred to species "4th09". Distance-matrices were constructed with our method from the resulting genomes, and trees were constructed using the BioNJ method (Supplemental Fig. 4e).

6. Artificial lateral gene transfer between two organisms

E. coli 536 (~4,600 ORFs) was used as an acceptor data set, and either *Salmonella enterica* subsp. *enterica* serovar Typhi str. Ty2 (close species: ~4,300 ORFs) or *Synechocystis* sp. PCC 6803 (distant species: ~3,600 ORFs) was used as the donor dataset. From the donor data set, either ~460 or ~1,380 ORFs (corresponding to 10% or 30% of ~4,600) were randomly selected, and added to the acceptor dataset. Then the distance-matrix of 188 species (*E. coli* 536 with 10% LGT, *E. coli* 536 with 30% LGT, and the other 186 species) was constructed based on our method. The tree was constructed using the BioNJ method (Supplemental Fig. 4c and 4d) as described above.

Supplementary Text 1

1. Bacteria topology

Almost all the organisms within the same phyla form single clades, with a few exceptions (Fig. 2). Our whole-proteome phylogenetic tree contains three classes of Actinobacteria, Actinobacteridae (60 species), Rubrobacteridae (1 species), and Coriobacteridae (1 species). All the Actinobacteridae species form a single clade, and the phylogenetic relationships in this clade (Fig. 3, Supplementary Fig. 2, page 2) are largely consistent with a previous report [55]. Coriobacteridae is a very divergent Actinobacteria class [55], and deeply rooted in our tree, not from the within the Actinobacteria clade, but from within the clade containing Firmicutes and Fusobacteria.

Firmicutes forms a single clade with the exception of one species, *Coprothermobacter proteolyticus* DSM 5265, which does not cluster with other Firmicutes in our analysis. Although *Coprothermobacter proteolyticus* DSM 5265 has been assigned to Firmicutes, a recent report suggests that it may actually belong in the Dictyoglomi-Thermotogae-Aquificae group [56], which is consistent with our results. The remainder of the phylogenetic relationships in the Firmicutes cluster (Fig. 3, Supplementary Fig. 2, page 3) are largely consistent with previous reports [57,58].

Cyanobacteria and Chlamydiae each comprise separate clades in our tree (Fig. 3), the topologies of which are largely consistent with previous reports (Supplementary Fig. 2, page 4). Chlorobi and Bacteroidetes form a large clade, to the exclusion of all others in our analysis.

α-, ζ-, γ-, β-Proteobacteria form a single cluster, except for *Candidatus* Carsonella ruddii Pv, which does not cluster with other γ-Proteobacteria (Fig. 3). *Candidatus* Carsonella ruddii Pv, is the primary endosymbiont of the psyllid *Pachypsylla venusta*, and possesses only 182 predicted open reading frames (ORFs) [59]. Most δ-Proteobacteria and all ε-Proteobacteria form a clade with Aquificae, Nitrospirae, Elusimicrobia, and Chloroflexi. Myxococcales and Bdellovibrionales are not placed in this clade by our method, even though they are classified as δ-Proteobacteria. Aquificales has been proposed as one of the deepest lineages in the bacterial tree [60,61]; however, a close relation has also been reported between Aquificales and ε-Proteobacteria [62], which is consistent with our tree. The true position of Aquificales is still uncertain, and further studies are required. Nitrospirae has also been suggested as being closely related to δ-Proteobacteria [63], which is largely consistent with our tree. Elusimicrobia deeply branched from within the Proteobacteria, which is quite different from other reports [64]. What properties of the Elusimicrobia genome produce this topology is not evident.

The entangled structure of the origin of Bacteria may be due to the diversity of δ-Proteobacteria species and/or methods of tree reconstruction. Supplementary Fig. 5 shows a consensus tree constructed from 100 trees of 144 species randomly selected from each order. Some branches around

Proteobacteria show lower consensus values than 50% (dotted lines), indicating that a large number of Proteobacteria species belong to the different orders in our tree. We note that some orders, including Myxococcales and Bdellovibrionales, branch out at different positions depending on whether the analysis had been performed with the Fitch–Margoliash least-squares or NJ method [54]. In the Fitch–Margoliash method the δ-Proteobacteria orders are placed with other δ-and ε-Proteobacteria. However, in the NJ method Myxococcales is placed near Actinobacteria and Chloroflexi (data not shown). In summary, the Fitch–Margoliash method yielded topologies most consistent with accepted taxonomy. Unfortunately, the Fitch–Margoliash program distributed in the PHYLIP package (ver. 3.69) [54] uses a single core, and requires an extraordinary amount of calculation time (one year for 721 species, shown in Supplementary Fig. 1g), and could not be used for the 1,087 species in our study.

2. Eucarya topology

The phylogenetic relationships among eukaryotes that our method reconstructed are largely consistent with current understanding [65]. Fungi and Metazoa form one clade and Amoebozoa and a parasitic fungus (*Encephalitozoon cuniculi*) form another clade, both branching from a deep common ancestor (Fig. 2). The Viridiplantae and Rhodophyta topology (Supplementary Fig. 2, page 1), as sister groups to each other, is consistent with previous report [66]. Alveolata and Stramenopiles do not form a single clade, as suggested by other reports [67], but are paraphyletic in our tree, deeply branching in the base of the eukaryotic tree. Interestingly, *Encephalitozoon cuniculi* GB-M1, a fungus, does not fall into the main Fungi clade in our analysis, perhaps due to its extremely small genome size (~2.9 Mb) [68]. Rather, *E. cuniculi* GB-M1 branches deep in the eukaryote clade in our tree, consistent with a P_5 P-type ATPase tree [69]. Branching pattern of Fungi, Animals, Amoenozoa, Stramenopiles and Archaeplastida is well consistent with recent tree from a global phylogenomic perspective [66].

3. Archaea topology

Two major Archaea phyla, the Crenarchaeota and the Euryarchaeota, were initially designated. Recently, two other phyla have been proposed, the Aigarchaeota and Korarchaeota [70]. Evolutionary relationships of these groups have been extensively studied, using many approaches. Phylogenies based on rRNA indicate that Crenarchaeota evolved from Euryarchaeota, but a phylogenetic tree reconstructed from 38 concatenated marker genes indicates that Crenarchaeota and Euryarchaeota diverged directly from the root of archaeal evolution [70]. Our whole-proteome phylogenetic tree, with paraphyletic archaeal phyla (Fig. 3, Supplementary Fig. 2, page 1), is similar to a ribosomal protein tree [37].

We examined the topology of the class Methanomicrobia next. Methanomicrobia consists of three methanogen orders, whose phylogeny has been difficult to fully resolve. Methanomicrobia

contains three clades in our phylogeny (Fig. 2, Supplementary Fig. 2, page 1), with *Archaeoglobus fulgidus* DSM 4304 serving as outgroup; the phylogenetic relationships within each clade are the same as previously reported [71].

Nanoarchaeum equitans has the smallest genome in Archaea [30], and is parasitic on another archaeon [72]. This organism appears to have diverged from the archaeon tree earlier than Crenarchaeota [30], which is consistent with our tree. However, *N. equitans* appears embedded within the Euyarchaeota in our analysis, which is different from other phylogenies [30].

4. Phylogenetic analysis of the *E. coli/Shigella* cluster

We next examined the power of our approach to resolve closely related organismal relationships. For this purpose, we evaluated the branching pattern of the *E. coli/Shigella* cluster in our analysis (Supplementary Fig. 2, page 5). A large number of *E. coli/Shigella* strains have been sampled and their genome sequences have been reported. Whole-genome sequences enable detailed phylogenetic analyses of these organisms, and many hypotheses regarding the phylogenetic relationships among the *E. coli/Shigella* strains have been proposed [73]. *E. coli* strains are classified into several phylogroups, such as A, B1, B2, D, and E. The members of each group within our study are identical to previous reports, except that *E. coli* 53638, which is a member of group A [74], falls into group B1 in our tree. According to our phylogenetic tree, the B2 group branches most deeply, followed by the appearance of groups E and D. Groups B1 and A appear most recently. This topology is similar to previous reports [75].

Shigella is a pathogenic bacterium that is very closely related to *E. coli*. Two different evolutionary scenarios have been proposed for the origin of *Shigella* [76,77]. One scenario involves a plural *Shigella* origin from several *E. coli* strains, with each acquiring a virulence plasmid independently. The other is a single-ancestor scenario. According to this scenario, one *E. coli* strain evolved into *Shigella*, and all subsequent *Shigella* strains form a single clade. In our analysis *E. coli* and *Shigella* separate in the initial stages of *E. coli/Shigella* evolution, followed by diversification into the various *E. coli* phylogroups, and all the *Shigella* strains examined form a single clade. This suggests the second scenario, where *Shigella* evolved from a single ancestral *E. coli*. However, recent studies using core genes have shown the genus *Shigella* split into several clusters interspersed in the *E. coli/Shigella* phylogeny, which supports the first, multiple-origin scenario with Shigella emerging from several *E. coli* ancestors [78,79]. Regardless, phylogenetic relationships within the *Shigella* clade in our analysis are consistent with a previous report [73]. *Escherichia fergusonii*, which causes disease in animals, is an outgroup to the entire *E. coli/Shigella* cluster in another previous report [80], and this also concurs with our analysis.

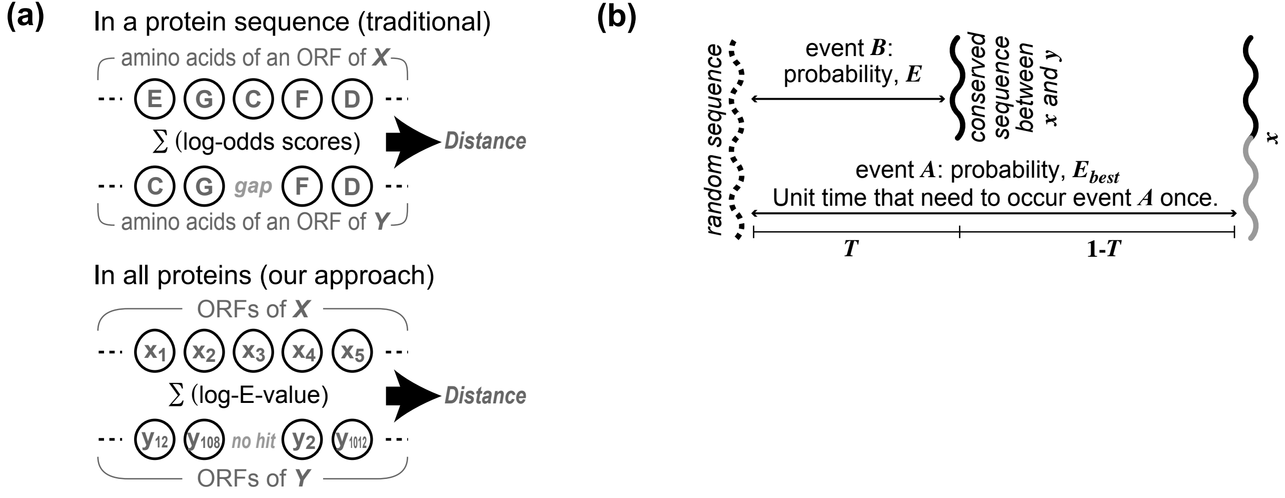

Fig.S1 page 1

(a) Schematic similarity between traditional approach and our approach to estimate the distance.

(b) Relationship between ***T*** and probabilities. 1-***T*** reflects relative time necessary to ***X***'s acquiring additional sequence in ORF ***x*** (gray line) after diversion of ***X*** from common ancestor of ***X*** and ***Y***.

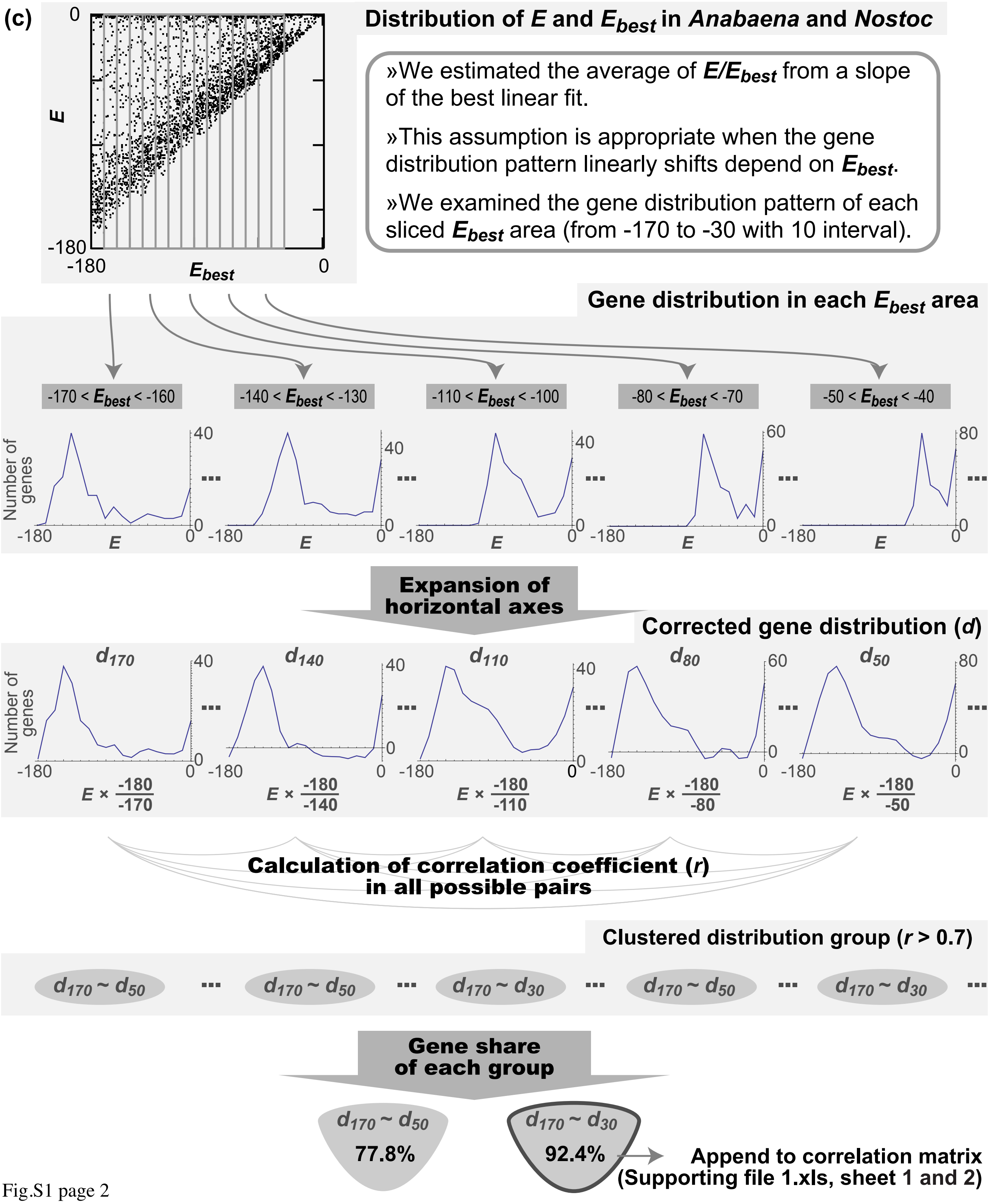

Fig.S1 page 2

(c) Analysis of the distribution profile of e-value (***E***) on a two dimensional graph. ***E*** and ***E$_{best}$*** were calculated for all the combinations of 187 species used in Fig S4a or 1087 species used in Fig. 2. Plots on a graph were divided into 14 groups corresponding to ***E$_{best}$*** (-170 ~ -160, -160 ~ -150, -150 ~ -140, -140 ~ -130, -130 ~ -120, -120 ~ -110, -110 ~ -100, -100 ~ -90, -90 ~ -80, -80 ~ -70,. -70 ~ -60, -60 ~ -50, -50 ~ -40, and -40 ~ -30). For each group, the plots were further divided into 18 groups corresponding ***E*** (-180 ~ -170,-170 ~ -160, -160 ~ -150, -150 ~ -140, -140 ~ -130, -130 ~ -120, -120 ~ -110, -110 ~ -100, -100 ~ -90, -90 ~ -80, -80 ~ -70,. -70 ~ -60, -60 ~ -50, -50 ~ -40, -40 ~ -30, -30 ~ -20, -20 ~ -10, and -10 ~ 0). A histogram of gene number was plotted against ***E***. Then, horizontal axis was expanded as in Fig. S1c (d, corrected gene distribution). Correlation coefficient was calculated between all possible pairs of 14 groups, and the groups were clustered with a threshold, r = 0.7. A cluster that contained the largest number of genes was selected as a representative distribution, and percentage of total genes in the cluster to all genes in the 14 groups was calculated. In most of the combinations in 187 or 1087 species (97%), more than 90% genes shows similar distribution pattern, suggesting adequacy of linear fit to estimate the average of ***E/E$_{best}$***. The percent of the genes belong to the largest cluster for 187 and 1087 species were listed in sheet 1 and 2 of supporting file 1.xls, respectively.

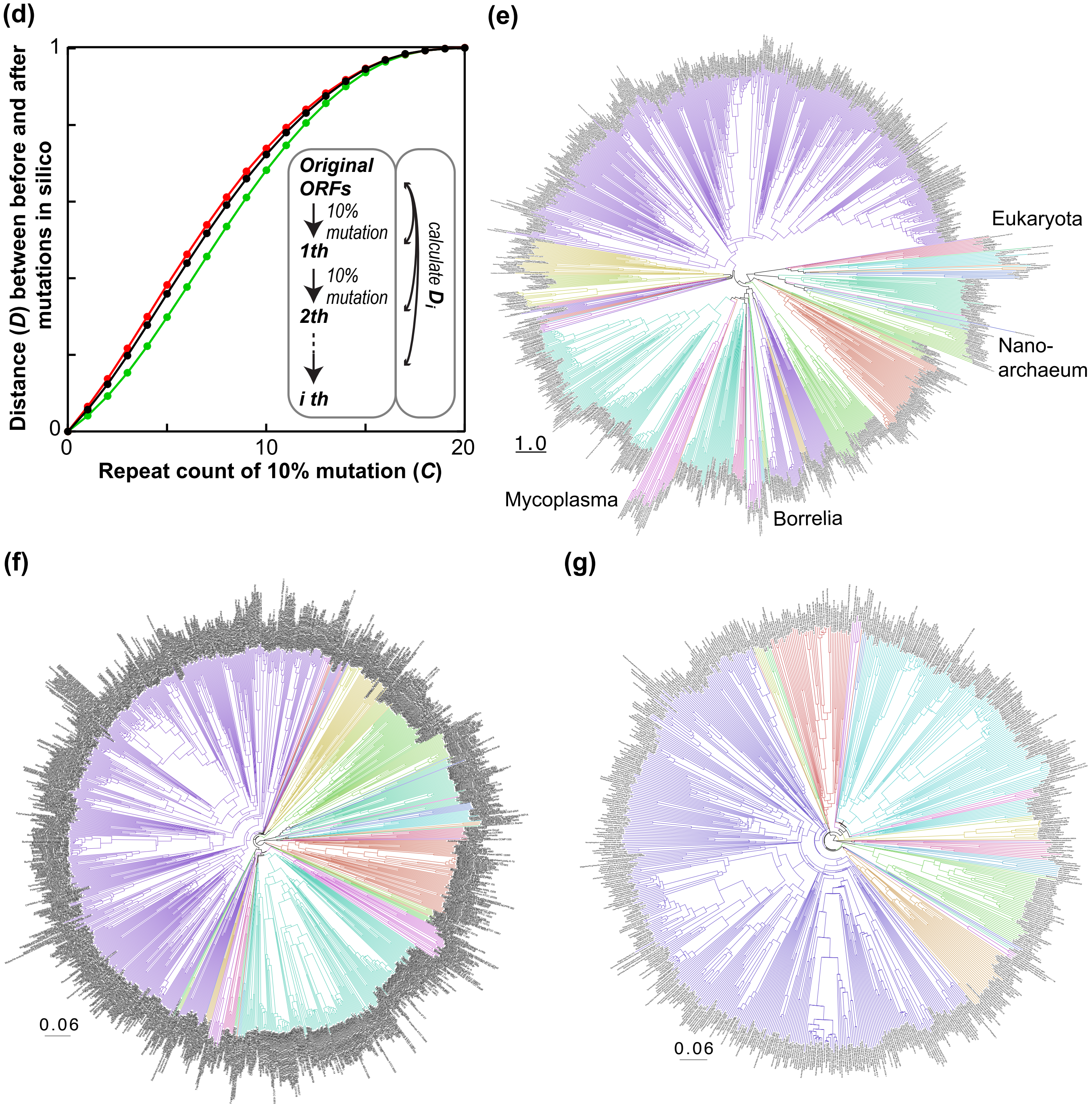

Fig.S1 page 3

(d) Simulation of distance saturation of all ORFs of each organism. In each organism, 10% of amino acids were mutated in one generation, and the distance from its origin was calculated (inset). Distances were plotted versus mutation time, and we obtained the saturation curve of each organism. Example of the curves were shown in this figure, where *Halobacterium salinarum NRC-1* (red), *Escherichia coli 536* (black), and *Arabidopsis thaliana* (green). Larger genome size slightly delayed the saturation.

(e) Tree constructed with distance after saturation correction ***C***. Basic structure was similar to the tree without correction (Fig. 2), however, some species showed longer branch length than other species. They are commensal species or they has histories of endosymbiotic event.

(f) Phylogenetic tree of 1087 species constructed based on the comparison of all protein sequences with Neighbor-Joining method.

(g) Phylogenetic tree of 721 species constructed based on the comparison of all protein sequences with Fitch-Margoliash method. Branch color reflects taxonomic information (division) obtained from NCBI website.

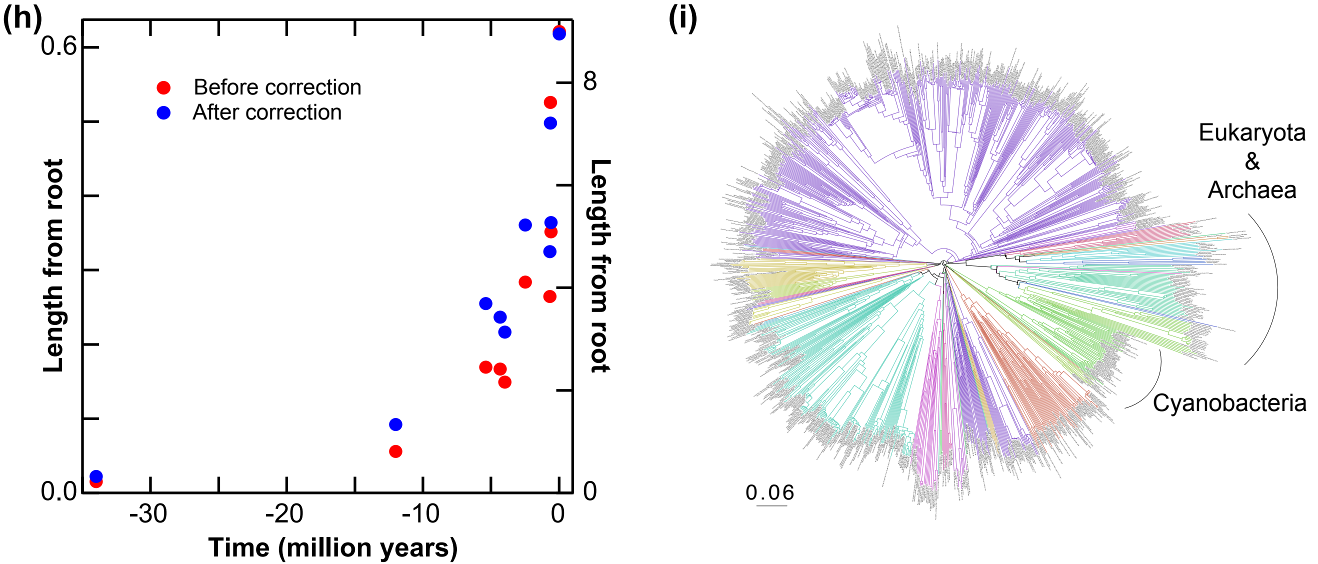

Fig.S1 page 4

(h) Comparison of branch length and actual time based on fossil records.

(i) Phylogenetic tree of 1087 species constructed based on the comparison of reciprocal pairs with BioNJ method.

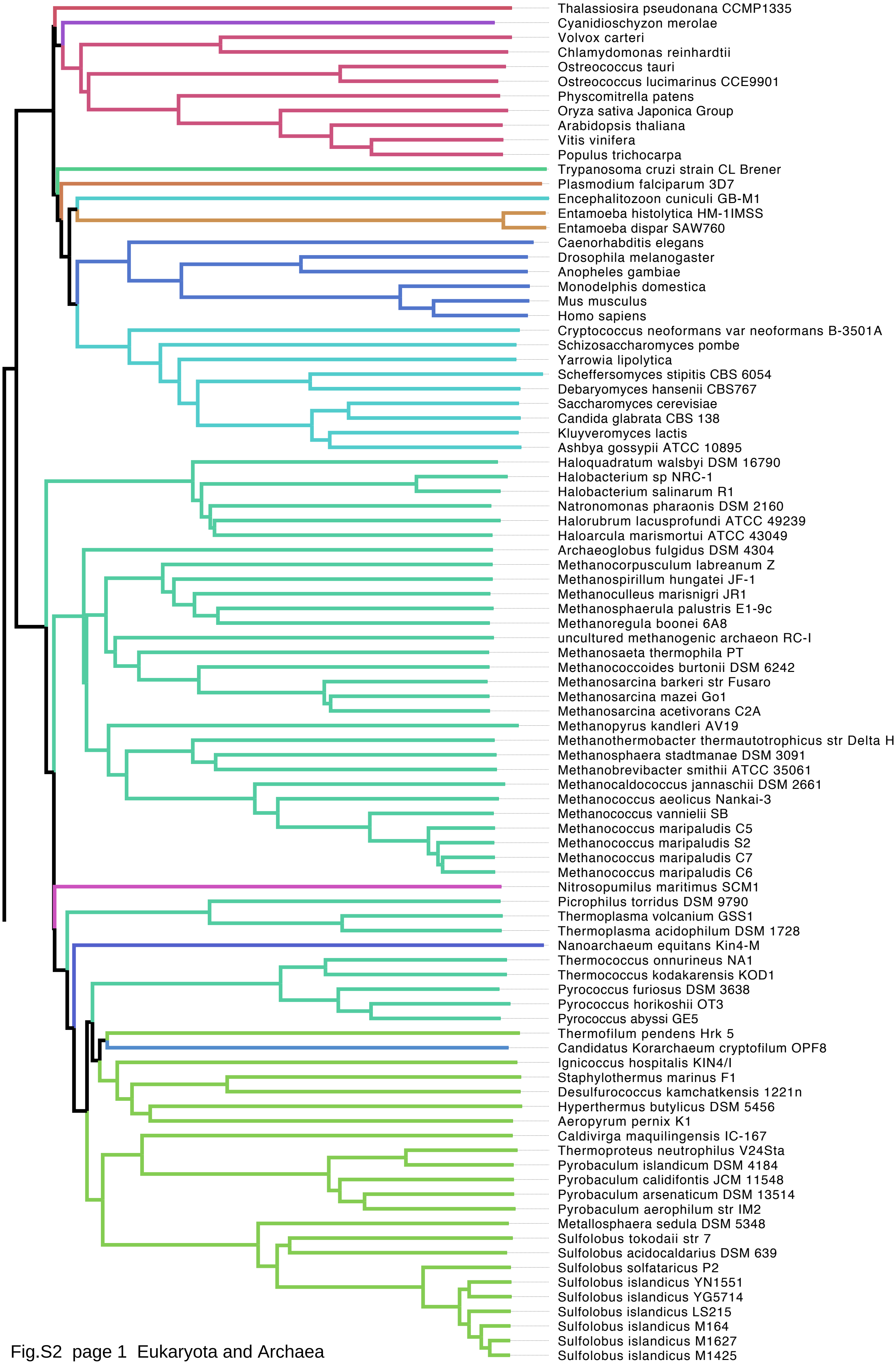

Fig.S2 page 1 Eukaryota and Archaea

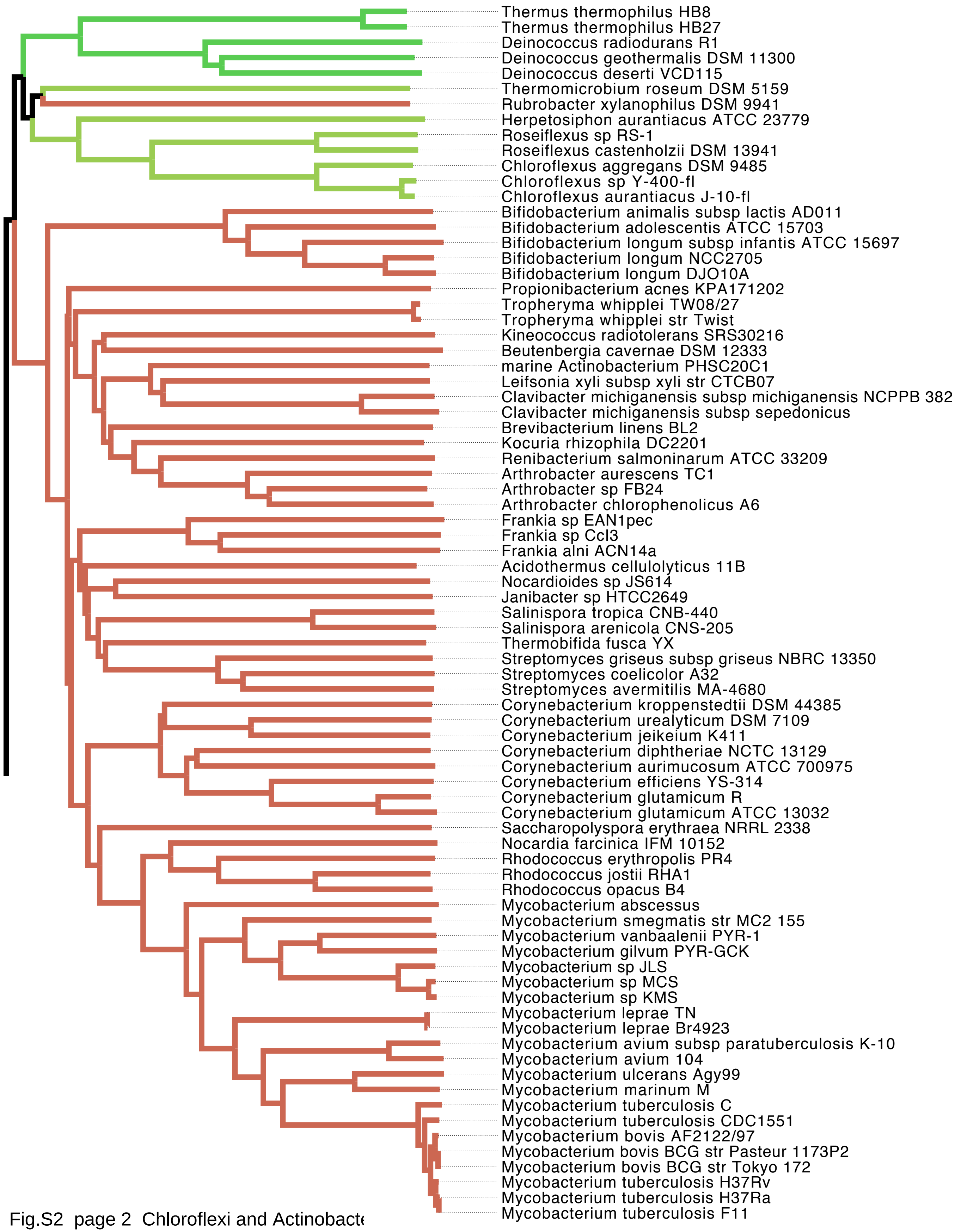

Fig.S2 page 2 Chloroflexi and Actinobacte

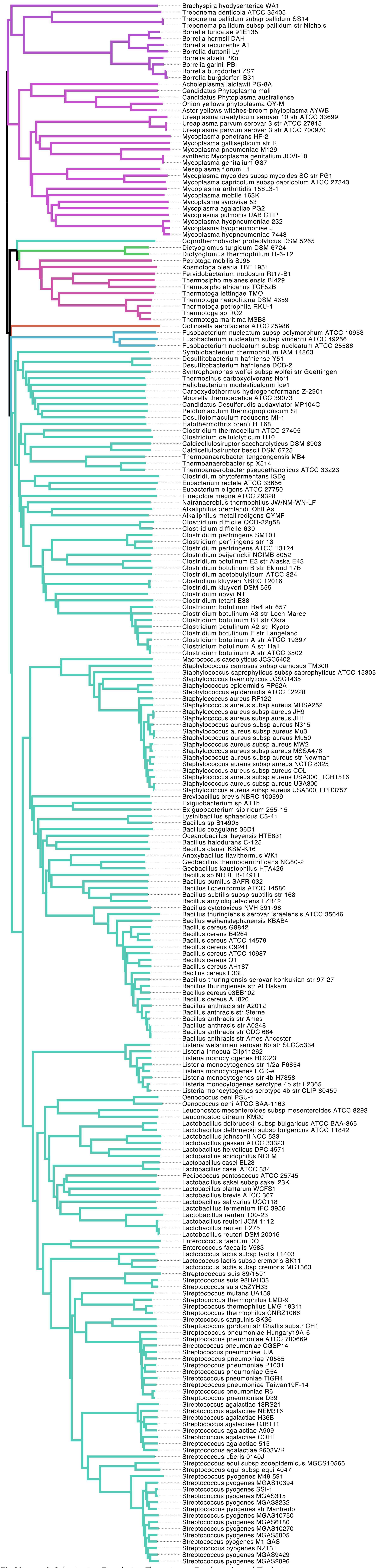

Fig.S2 page 3 Spirochaetes, Tenericutes, Thermotogae, Fusobacteria, and Firmicutes

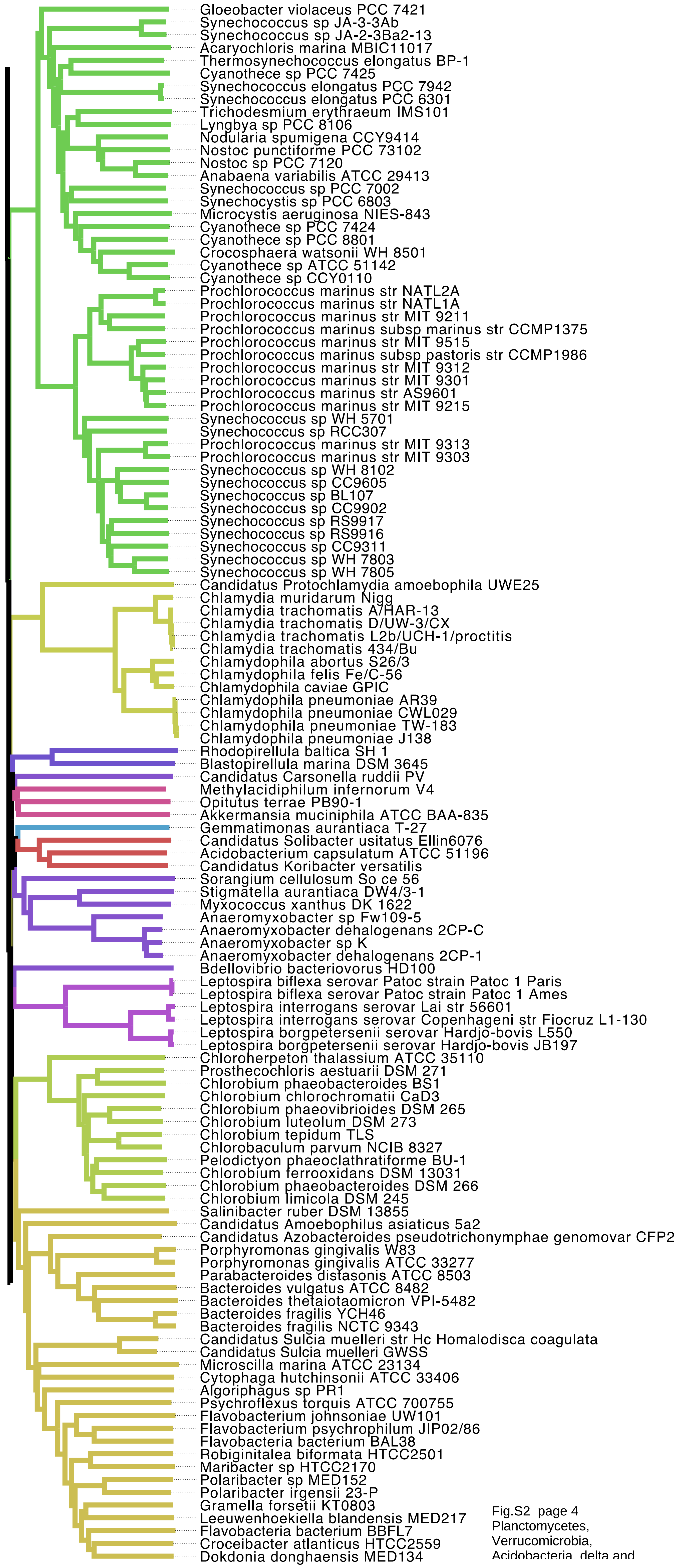

Fig.S2 page 4
Planctomycetes,
Verrucomicrobia,
Acidobacteria, delta and

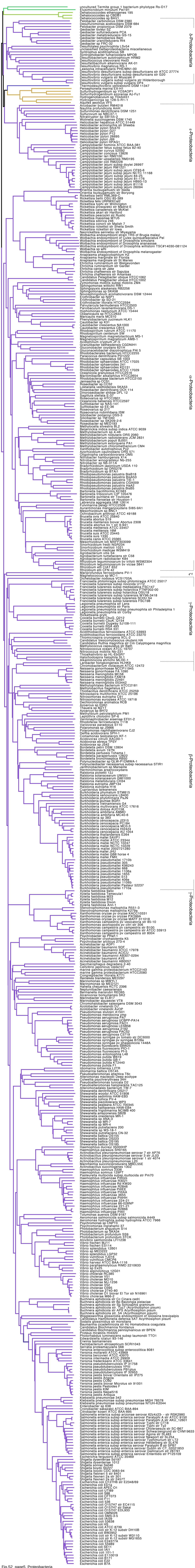

Fig.S2 page5 Proteobacteria

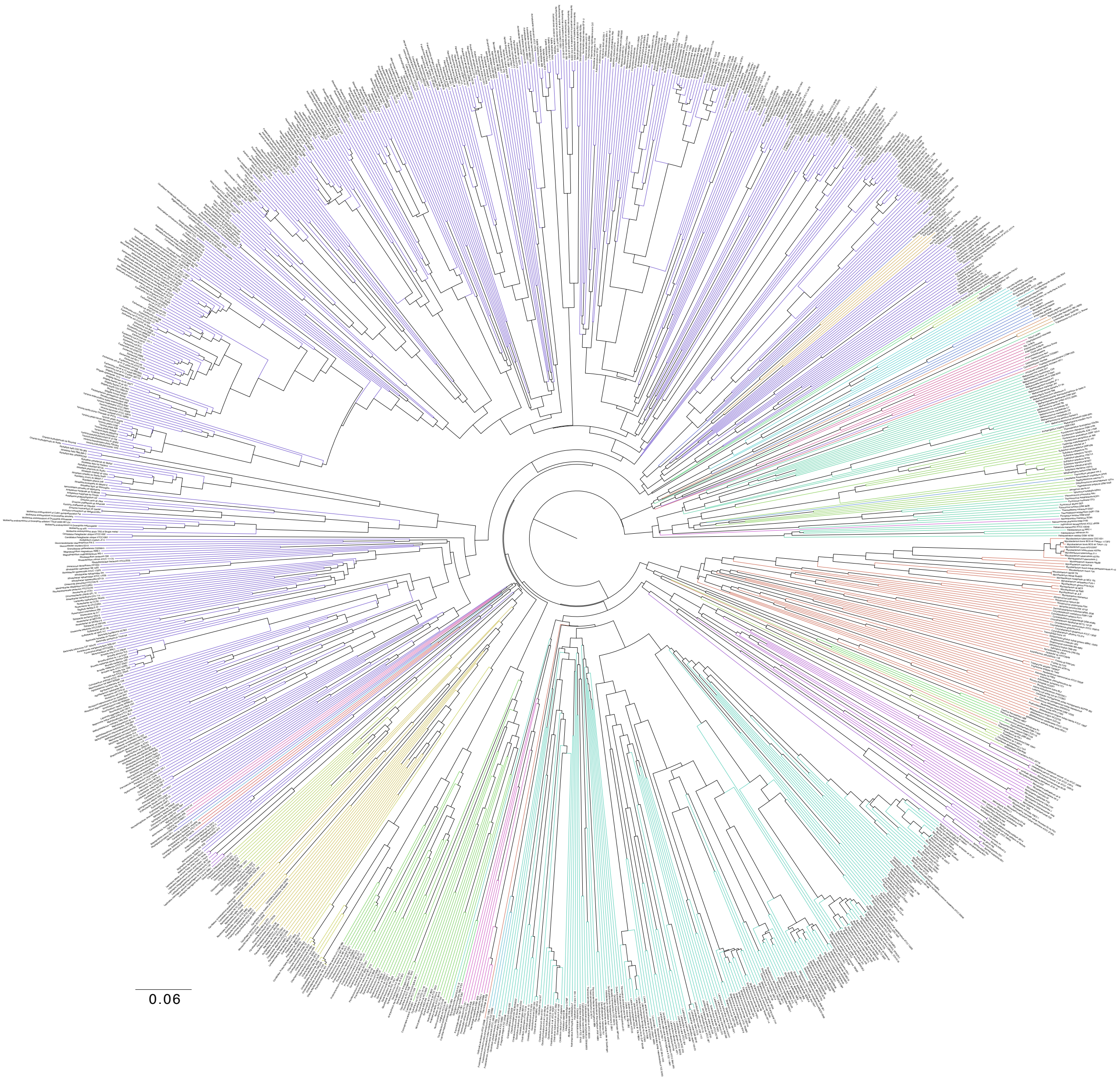

Fig. S3
Phylogenetic tree of 1,085 species (excluding 2 parasites) reconstructed from a comparison of all protein sequences. Branch colors reflect taxonomic information (division) obtained from the NCBI Website.

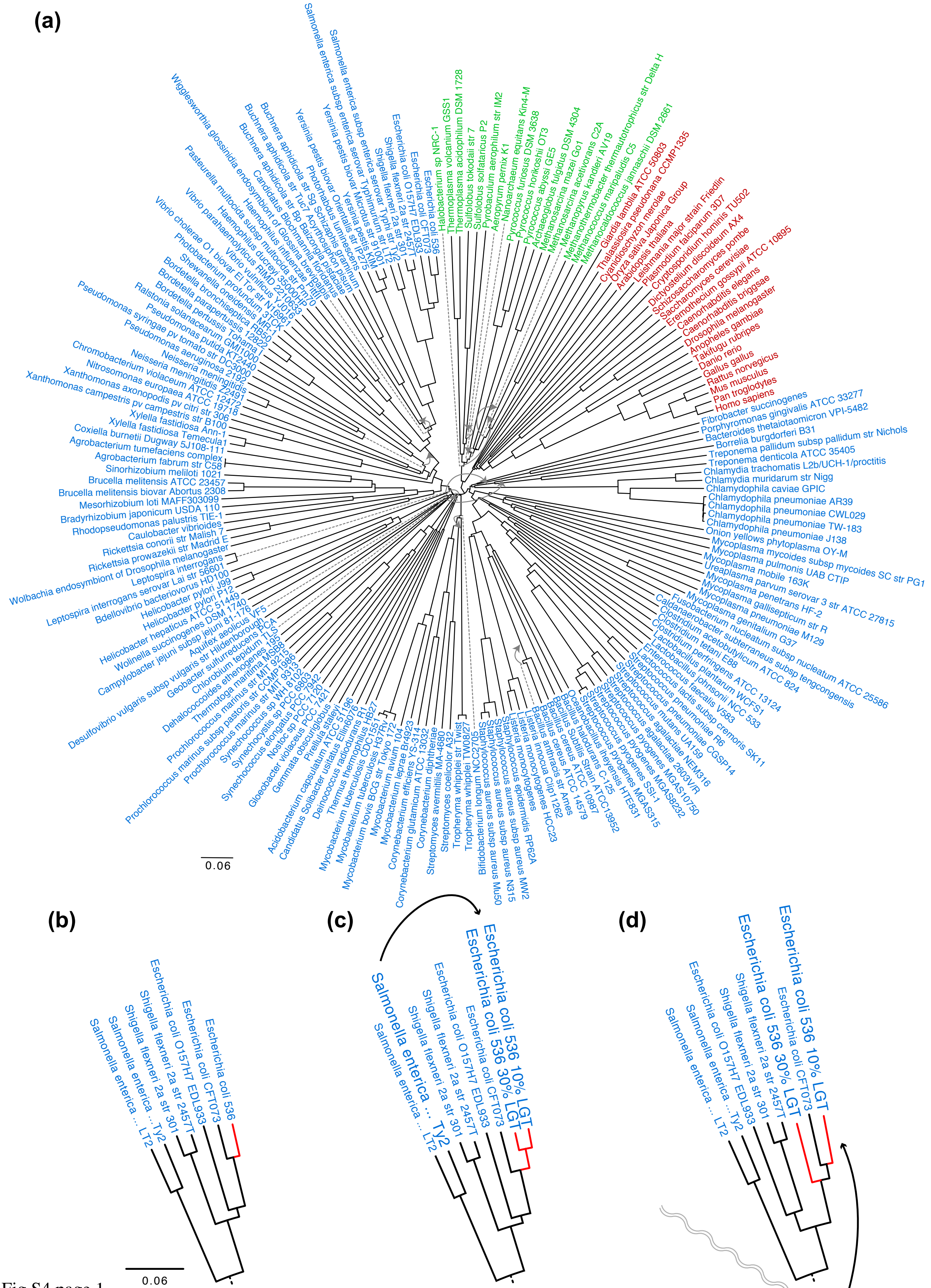

Fig.S4 page 1

(a) Comparison of the tree topology constructed by the present method with that by ML method. Distance-matrix for 187 species (Fig. 2 and Table S4 in Ciccarelli et al. 2006) was constructed based on our method, and the tree was constructed by BioNJ method. The branching patters of our tree were compared to those by the Ciccarelli' s tree which were supported by 80-100% bootstrap values. Different branches from Ciccarelli' s tree were marked with gray dotted lines. Arrow heads indicate the branching position in Ciccarelli' s tree.

(b,c,d) Effect of lateral gene transfer on the tree topology of 187 species. The randomly selected *Salmonella* **(c)** or *Synechocystis* **(d)** genes corresponding to 10 or 30 % of gene number of *Escherichia coli* 536 were artificially transferred to *Escherichia coli* 536 (Escherichia coli 536 10% LGT and Escherichia coli 536 30% LGT, respectively). The LGT from *Salmonella* did not affect the branching pattern. The LGT from *Synechocystis* did not affect the position of the *Escherichia coli* 536, but its neighboring species (Escherichia coli O157H7 EDL933) was slightly affected. Other species showed the same branching pattern to Fig. S4a. **(b)** Enlarged view of Fig. S4a around *Escherichia coli* 536.

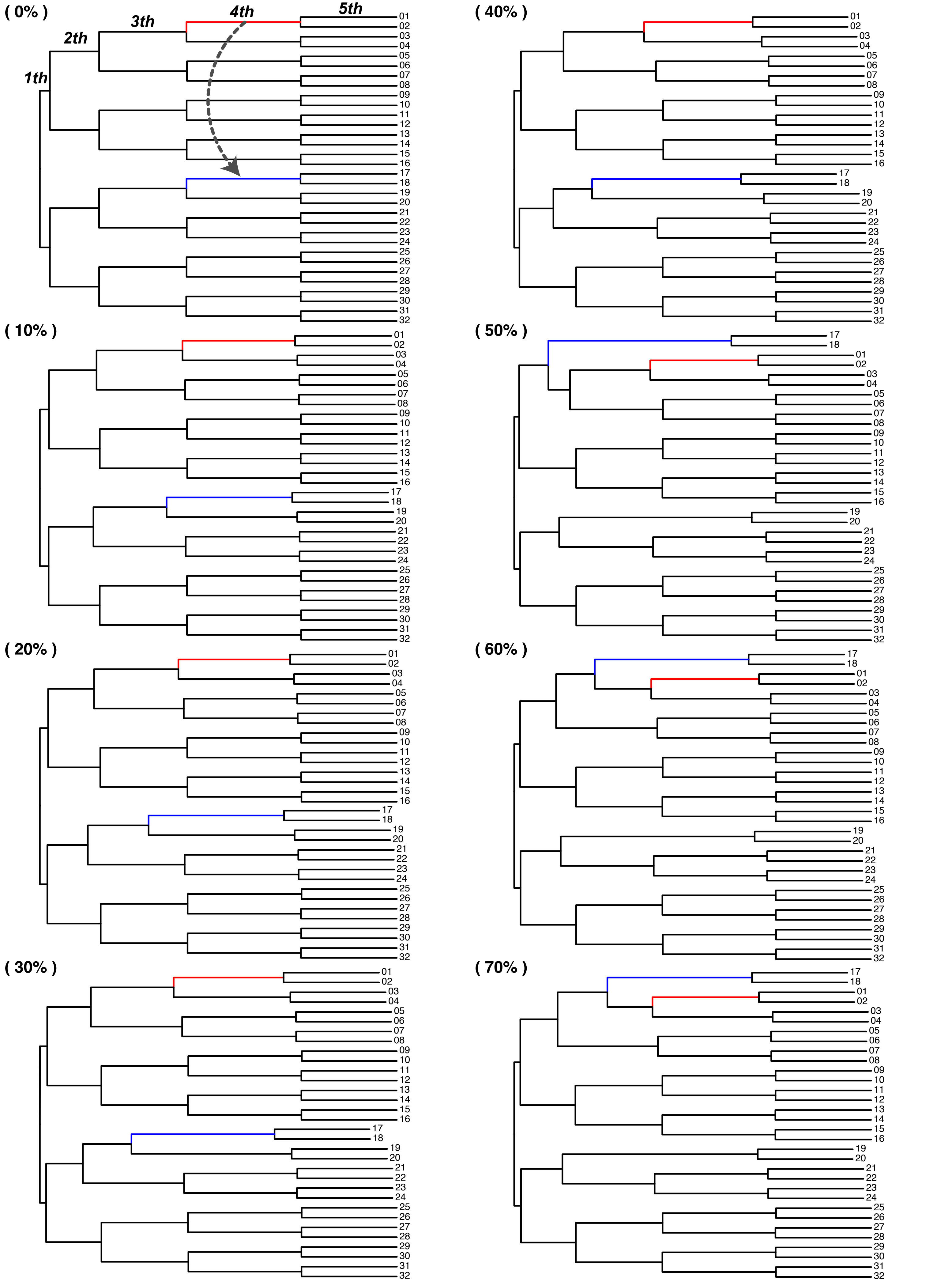

Fig.S4 page 2

(e) Effect of lateral gene transfer on the tree topology of in silico evolved *Escherichia coli* 536. At 4th generation, 0, 10, 20, 30, 40, 50, 60, or 70% genes was transferred from red to blue. Other parameters were the same to Fig. 1b. Up to 40%, the branching pattern was not affected.

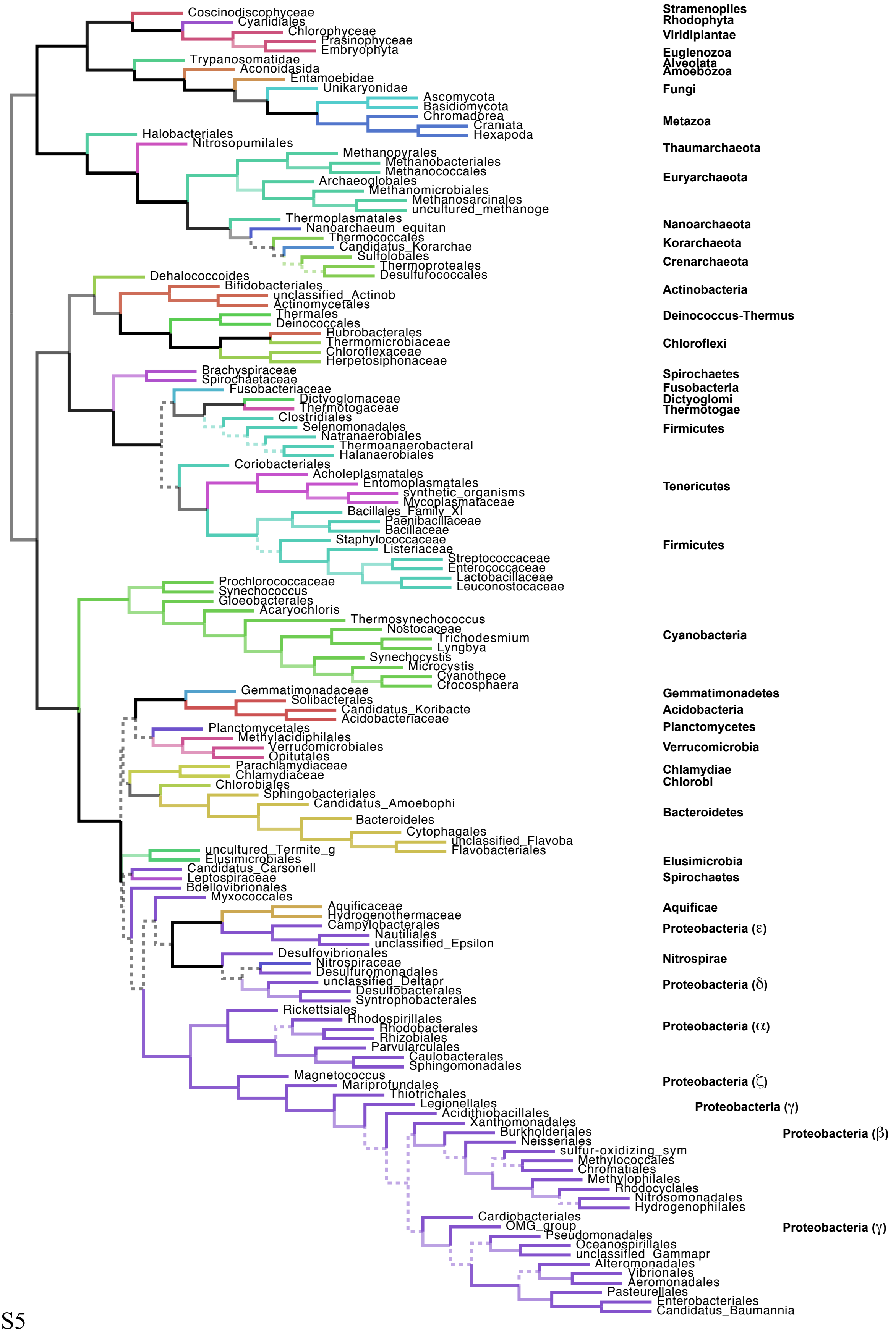

Fig. S5

Majority-rule consensus tree reconstructed from random representative species from 144 orders. Lower transparency in branch color reflects higher frequency, and dotted line indicates < 50% frequency.
